# Effects of the non-pharmacological interventions of traditional Chinese medicine on postpartum depression: A protocol for systematic review and network meta-analysis

**DOI:** 10.1097/MD.0000000000028939

**Published:** 2022-03-04

**Authors:** XiaoMei Huang, Shu Luo, Hongwei Wang

**Affiliations:** 1 Department of Obstetrics, The Second Affiliated Hospital of Hainan Medical College, Haikou, Hainan, China.

**Keywords:** network meta-analysis, non-pharmacological interventions, postpartum depression, protocol, traditional Chinese medicine

## Abstract

**Background:**

Postpartum depression (PPD) has become one of the common disorders during the postpartum period. The non-pharmacological interventions of traditional Chinese medicine (TCM) have achieved good results in the treatment of PPD. However, the efficacy of different non-pharmacological interventions of TCM for PPD has not been fully elucidated. Due to the large number of non-pharmacological intervention of TCM modalities, the selection of appropriate non-pharmacological interventions of TCM has become an urgent clinical problem. The aim of this network meta-analysis was to explore the best choice for different non-pharmacological interventions of TCM for PPD.

**Methods:**

PubMed, Web of Science, Scopus, Cochrane Library, Embase, China Scientific Journal Database, China National Knowledge Infrastructure, Chinese Biomedical Literature Database, and Wanfang Data were searched to identify the randomized controlled trials of non-pharmacological interventions of TCM for PPD from the inception to February 2022. Two researchers will be independently responsible for literature screening, data extraction, and assessment of their quality. Standard pair-wise and Bayesian network meta-analysis will be performed to compare the efficacy of different non-pharmacological interventions of TCM for PPD via Stata 14.0 and WinBUGS1.4 software.

**Results:**

The results of this meta-analysis will be submitted to a peer-reviewed journal for publication.

**Conclusions:**

The conclusion of this systematic review will provide evidence for the selection of an optimal non-pharmacological interventions of TCM for PPD.

**Ethics and dissemination:**

Ethical approval is not required for this study. The systematic review will be published in a peer-reviewed journal, presented at conferences, and shared on social media platforms.

**OSF REGISTRATION NUMBER:**

DOI 10.17605/OSF.IO/TM96G.

## 1 Introduction

As the most common complication of childbirth, postpartum depression (PPD) is a serious and prevalent public health problem.^[1–3]^ According to the World Health Organization data, the probability of experiencing maternal mental disorders during pregnancy and postpartum is 10% and 13%, respectively, and is predominantly depression. In developing countries, the rates are even higher: 15.6% during pregnancy and 19.8% after delivery.^[4]^ According to 1 study, the detection rate of maternal PPD was 15%.^[5]^ When PPD is not treated in a timely manner, it not only harms the mother's body and mind, but also affects the cognitive function and intelligence of infants and children, which increase the risk of disease.^[6–8]^ Due to the high incidence of PPD and the wide range of risks, timely and accurate identification and treatment are particularly important.

At this stage of medical treatment, PPD is still mainly treated with medication, but its efficacy varies significantly among individuals, which is slow to take effect, with many side effects for mothers who need to breastfeed their babies. Therefore, non-pharmacological treatments are gradually coming into the public and are highly recognized by many health care professionals and patients with PPD for their high compliance, low side effects and significant efficacy.^[3,9–11]^

PPD does not belong to the category of “depression” in traditional Chinese medicine (TCM).^[12,13]^ The non-pharmacological interventions of TCM mainly include acupuncture, ear point pressing, acupoint embedding, Baduanjin, tai Chi, etc. Meanwhile, the non-pharmacological interventions of TCM for PPD have been widely developed.

There exists valid evidence confirming the definitive efficacy of multiple non-pharmacological interventions of TCM for the treatment of PPD.^[14,15]^ However, due to the wide variety of non-pharmacological interventions of TCM, the optimal non-pharmacological interventions of TCM for patients with PPD are unclear. To our knowledge, compared with the efficacy and safety of different non-pharmacological interventions of TCM, a network meta-analysis (NMA) has not been previously completed. To promote the rational selection of non-pharmacological interventions of TCM, improve safety, and provide an adequate evidence-based medical rationale, this study will conduct the NMA reporting randomized controlled trials (RCTs) of non-pharmacological interventions of TCM for PPD to help clinical staff choose the best option among different interventions.

## 2 Methods

### 2.1 Study registration

The protocol of this review was registered in OSF (OSF registration number: DOI 10.17605/OSF.IO/TM96G). Besides, it was reported as the statement guidelines of preferred reporting items for systematic reviews and meta-analysis protocol.^[16]^

### 2.2 Inclusion criteria for study selection

#### 2.2.1 Types of studies

The RCTs of non-pharmacological interventions of TCM for PPD will be included.

#### 2.2.2 Types of participants

Postpartum women aged ≥18 years and meeting the diagnostic criteria of depressive mental disorder or depression Score Scale.

#### 2.2.3 Types of interventions

The control group received standard nursing regimen. On this basis, the non-drug intervention of TCM was carried out in the experimental group, mainly including acupuncture, ear point bean-pressing, acupoint embedding, Baduanjin, tai chi, etc.

#### 2.2.4 Types of outcome indexes

The types of outcome indexes include Hamilton Depression Rating Scale (HAMD), Beck Depression Inventory (BDI), Self-Rating Depression Scale (SDS), and Edinburg Postpartum Depression Scale (EPDS).

#### 2.2.5 Exclusion criteria

- The literature data are incomplete, and the relevant data cannot be obtained by contacting the author;
- For repeated literatures, only the latest and most complete literatures were included.
- Editorials, letters, reviews, pharmacological or chemical experiments, etc.

### 2.3 Data sources

The RCTs of non-pharmacological interventions of TCM for PPD published before February 2022 will be systematically searched from PubMed, Web of Science, Scopus, Cochrane Library, Embase, China Scientific Journal Database, China National Knowledge Infrastructure, Chinese Biomedical Literature Database, and Wanfang Data. In addition, the unpublished related studies in the Clinical trial registry were searched and references to the included literature were traced to ensure the recall rates. The literature retrieval strategies are displayed in Table 1, with PubMed database as an example.

**Table 1:** Search strategy in PubMed database.

Number	Search terms
#1	Depression, Postpartum[MeSH]
#2	Postnatal Depression[Title/Abstract]
#3	Postpartum Depression[Title/Abstract]
#4	Post-Natal Depression[Title/Abstract]
#5	Post-Partum Depression[Title/Abstract]
#6	Depression, Post-Natal[Title/Abstract]
#7	Depression, Post-Partum[Title/Abstract]
#8	Depression, Postnatal[Title/Abstract]
#9	Post Natal Depression[Title/Abstract]
#10	Post Partum Depression[Title/Abstract]
#11	or/1-10
#12	Medicine, Chinese Traditional[MeSH]
#13	Chinese Medicine, Traditional[Title/Abstract]
#14	Chung I Hsueh[Title/Abstract]
#15	Traditional Medicine, Chinese[Title/Abstract]
#16	Zhong Yi Xue[Title/Abstract]
#17	Chinese Traditional Medicine[Title/Abstract]
#18	Traditional Chinese Medicine[Title/Abstract]
#19	Hsueh, Chung I[Title/Abstract]
#20	or/12–19
#21	Randomized Controlled Trial[MeSH]
#22	Random∗[Title/Abstract]
#23	Clinic trial [Title/Abstract]
#24	or/21-23
#25	#11 and #20 and #24

### 2.4 Data collection and analysis

#### 2.4.1 Data extraction and management

**Figure 1:**
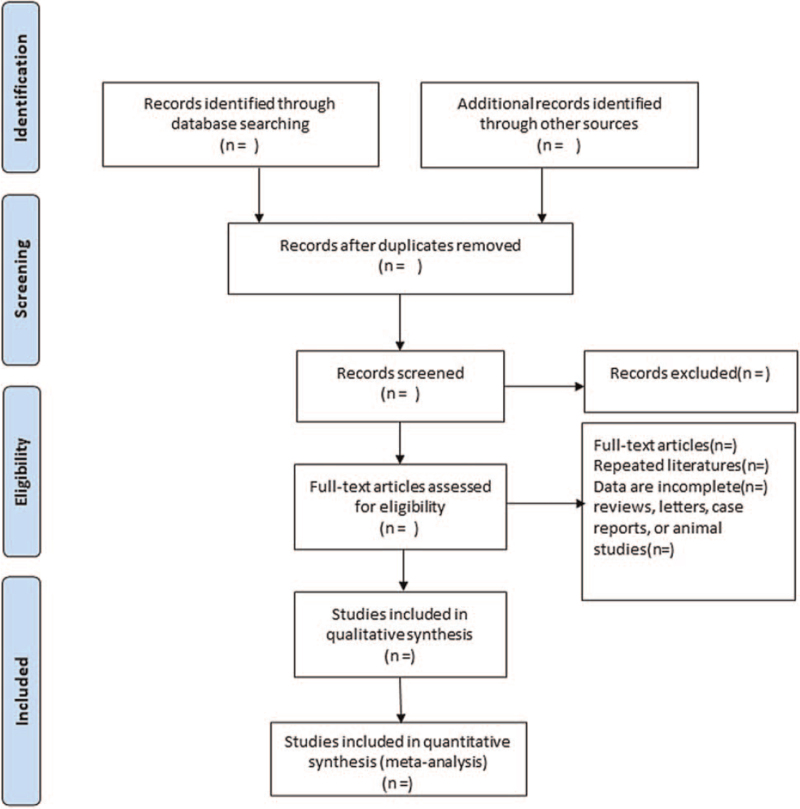
Flow diagram of study selection process.

The literature was screened by 2 evaluators separately. First, the literatures that were duplicated and did not meet the inclusion criteria were eliminated by reading the text title and abstract. Second, the literatures that could be included were further read in full and cross-checked for results. For the literatures with disagreement, 2 evaluators discussed and negotiated together. If agreement could not be reached, a third evaluator was asked to further evaluate the literatures. The following data were extracted from the included literatures: general information, such as title, author, country, and time of publication; study characteristics, such as age, sample size, intervention, follow-up time, and number of missed visits; outcome indicators; the risk of bias evaluation details. The screening flow chart of this study is presented in Figure 1.

#### 2.4.2 Assessment of the risk of bias

The quality of the literatures was evaluated using the RCT bias risk assessment tool recommended in Cochrane Handbook 5.1.0.^[17]^ The evaluation results will be classified into the high-risk, low-risk, and unclear categories.

#### 2.4.3 Measures of treatment effect

Continuous variables will be combined using the standardized mean differences and corresponding 95% confidence intervals.

#### 2.4.4 Management of missing data

In case of any missing data in relevant studies, the original data will be requested by email. If there is a failure in the data request, such data shall be excluded from this study.

#### 2.4.5 Assessment of heterogeneity and data synthesis

The heterogeneity will be assessed by the *I* ^2^ test. *I* ^2^≤50% will be considered as a small heterogeneity, and a fixed-effect model will be used to combine the effect size. Otherwise, a random-effects model will be introduced. The consistency of direct comparison and indirect comparison will be judged by node splitting analysis in cases with closed rings. *P* > .05 suggests that there are no significant differences between the direct and indirect comparison, and a consistency model will be used. Otherwise, the inconsistent model will be adopted. Stata14.0 (STATA Corporation, College Station, TX) software will be used for direct comparison, and network evidence plots will be drawn. WinBUGS1.4 (MRC Biostatistics Unit, Cambridge, UK) software will be used for NMA, and Bayesian method will be used to present the possibility of each intervention by ranking probability.

#### 2.4.6 Assessment of reporting biases

A comparison-adjusted funnel plot will be depicted to identify the small sample effects between studies to assess the publication bias.^[18]^

#### 2.4.7 Subgroup analysis

The subgroup analysis will be conducted through intervention time.

#### 2.4.8 Sensitivity analysis

The sensitivity analysis will be conducted to assess the reliability by excluding each study each time and calculating the remaining.

#### 2.4.9 Ethics and dissemination

The contents of this paper do not involve moral approval or ethical review and will be presented in print or at relevant conferences.

## 3 Discussion

PPD leads to cognitive, behavioral, and emotional impairment in mothers, with long-term negative effects on their children. Some researches proved that few women come forward to seek professional help, and the barriers include feelings of stigma, childcare difficulties, lack of knowledge, financial constraints, lack of transportation, and lack of time.^[19]^ Besides, non-pharmacological interventions are more popular than medication among postpartum women due to concerns about medication side effects.^[20–27]^

However, there is no NMA on the comparative efficacy and safety of different non-pharmacological interventions of TCM for PPD. NMA can quantify and analyze different interventions for the treatment of the same disorder and rank all interventions, thus helping determine the best intervention. This study will summarize and rank the efficacy and safety of non-pharmacological interventions of TCM for PPD by NMA, which will provide a reference for determining the best non-pharmacological intervention of TCM.

## Author contributions

**Data collection:** Shu Luo.

**Funding support:** Hongwei Wang.

**Resources:** Shu Luo.

**Software operating:** Shu Luo.

**Supervision:** Hongwei Wang.

**Writing – original draft:** XiaoMei Huang and Hongwei Wang.

**Writing – review & editing:** XiaoMei Huang and Hongwei Wang.
